# Identification of *Streptococcus pneumoniae* and other Mitis streptococci: importance of molecular methods

**DOI:** 10.1007/s10096-020-03991-9

**Published:** 2020-07-24

**Authors:** Ewa Sadowy, Waleria Hryniewicz

**Affiliations:** 1 Department of Molecular Microbiology, National Medicines Institute, Chełmska 30/34, 00-725 Warsaw, Poland; 2 Department of Epidemiology and Clinical Microbiology, National Medicines Institute, Chełmska 30/34, 00-725 Warsaw, Poland

**Keywords:** *Streptococcus*, PCR, Sequencing, MLSA, WGS

## Abstract

The Mitis group of streptococci includes an important human pathogen, *Streptococcus pneumoniae* (pneumococcus) and about 20 other related species with much lower pathogenicity. In clinical practice, some representatives of these species, especially *Streptococcus pseudopneumoniae* and *Streptococcus mitis*, are sometimes mistaken for *S. pneumoniae* based on the results of classical microbiological methods, such as optochin susceptibility and bile solubility. Several various molecular approaches that address the issue of correct identification of pneumococci and other Mitis streptococci have been proposed and are discussed in this review, including PCR- and gene sequencing-based tests as well as new developments in the genomic field that represents an important advance in our understanding of relationships within the Mitis group.

## Introduction

The group of Mitis streptococci, included among Viridans streptococci (VGS) [1], includes currently about different 20 species [2, 3], which is found in humans and animals. Human Mitis streptococci significantly differ in their epidemiological importance. The best-known and most studied member of the group, *Streptococcus pneumoniae* (pneumococcus), commonly colonizes human nasopharynx but is also a very important etiologic agent of the respiratory tract and invasive infections. In the case of the community-acquired pneumonia, pneumococcus represents the most common pathogen and it was estimated that pneumococcal pneumonia caused 1.5 million deaths worldwide in 2015 including about 400,000 deaths in children younger than 5 years [4]. Other infections, especially pneumococcal bacteremia and meningitis, although less common, result in high mortality and long-term neurological sequelae in survivors. While improvements in treatment reduced the case-fatality ratio of pneumococcal meningitis, it still reached 25–27% in Europe and the Americas in 2015 and was as high as 61% in Africa in the same year [5]. Children and the elderly as well as immunocompromised patients, including HIV-infected people, constitute groups, which are most vulnerable to pneumococcal infections [6]. The development of resistance to commonly used antimicrobials, seen worldwide, complicates the treatment of pneumococcal infections [7]. After the introduction of conjugated vaccines into the mass vaccination of children a significant decrease in the incidence of invasive and respiratory infections caused by serotypes targeted by vaccines was observed [4]. However, vaccination also resulted in a serotype replacement, i.e. increased proportion of pneumococci with non-vaccine serotypes in carriage and disease [8], demonstrating fast adaptation of this pathogen to the selective pressure exerted by vaccines. In consequence, the reduced incidence of pneumococcal meningitis due to vaccination might be only temporary [9]. Other Mitis streptococci are typically non-pathogenic colonizers of the human nasopharynx, where they can co-reside with *S. pneumoniae.* However, severe invasive infections, such as infective endocarditis and bacteremia caused by these bacteria are occasionally observed, mostly in immunocompromised patients [10]. Infective endocarditis is a rare but severe disease, associated with high mortality reaching 40% [11]. A significant proportion of this disease is caused by the VGS [11–13]. Neutropenic cancer patients have an increased risk of developing VGS bacteremia, mostly associated with *Streptococcus mitis*, which in a significant number of cases results in serious complications, such as viridans streptococcal shock syndrome (VSSS), with mortality reaching 40–100% in paediatric patients [10, 13]. Treatment of these infections is additionally complicated by high rates of antimicrobial resistance among these bacteria [10].

Several studies reported that some representatives of the Mitis group may be mistakenly identified as *S. pneumoniae*, especially in samples obtained from non-sterile body sites [14–26]. Although such situations are relatively infrequent, it has to be stressed that distinguishing between *S. pneumoniae* and other Mitis streptococci is important for the understanding of the pathogenicity of particular species as well as the correct evaluation of disease burden and antimicrobial resistance levels in pneumococci and other Mitis streptococci. Such biases, in particular an overestimation of resistance among *S. pneumoniae* due to misidentification of the other Mitis streptococci as pneumococcus, were reported, e.g. in the USA and Portugal [18, 21, 27]. The phenomenon of misidentification is due to both a common evolutionary origin of these organisms as well as a horizontal gene transfer (HGT) among streptococcal species, residing in the same ecological niche [28, 29]. The issue of correct identification was addressed by several molecular and other approaches and the purpose of this review is to provide an overview of methods proposed so far, their feasibility and performance in the identification of *S. pneumoniae* and other Mitis streptococci.

## Taxonomic position of Mitis streptococci

Bacteria included in the genus *Streptococcus* are facultatively anaerobic, Gram-positive cocci. Streptococci demonstrate different types of haemolysis on blood agar, which led to the distinction of beta- and non-beta-haemolytic (alpha and gamma, i.e. non-haemolytic) streptococci, corresponding to the pyogenic and non-pyogenic groups, the later called also the viridians group streptococci (VGS) [30]. Analyses of sequences of 16S rRNA genes split the VGS into five subgroups, including Anginosus, Mitis, Salivarius, Bovis (Equinus) and Mutans [31–33]. Later on, the Bovis group was separated from the VGS, and the Sanguinis group was added [1]. Recently, genome-based studies introduced yet another organization of the *Streptococcus* genus, encompassing two main clades, designated Mitis-Suis and Pyogenes-Equinus-Mutans and composed of 14 subclades. The Mitis clade included Anginosus, Pneumoniae, Parasanguinis and Gordonii subclades; the Pneumoniae subclade grouped *S. pneumoniae*, *Streptococcus pseudopneumoniae*, *S. mitis*, *Streptococcus oralis*, *Streptococcus infantis*, *Streptococcus peroris* and *Streptococcus timonensis* [3]. Another study on Mitis streptococci, based on core genomic analyses combined with phenotypic characterization, led to the distinction of eight clades, with the Mitis clade encompassing clusters of *S. pneumoniae*, *S. pseudopneumoniae* and *S. mitis*, the later one comprised of several divergent strains [2]. This brief overview demonstrates the complexity of taxonomic relationships among streptococci.

## Phenotypic methods and automated systems

The preliminary identification of a streptococcal isolate is based on the morphology of colonies growing on blood agar. Pneumococci present typical colonies with a button or mucoid appearance [34]. However, a study performed on a large collection of pneumococcal clinical isolates from the respiratory tract showed that about 24% of isolates do not demonstrate this feature [18]. Classical microbiological identification of *S. pneumoniae* relies on susceptibility to optochin (ethylhydrocupreine hydrochloride) and solubility in sodium deoxycholate (bile) [35]. However, optochin susceptible and/or bile soluble isolates of other species from the Mitis group were reported in several studies [14–26]. The observed optochin susceptibility was shown to arise by a presumable transfer of *atp* genes from *S. pneumoniae* [17]. At the same time, a small number of *S. pneumoniae* isolates demonstrate optochin resistance and/or poor bile solubility [23, 36, 37]. Nevertheless, determining optochin susceptibility and bile solubility remains the key test for phenotypic identification of *S. pneumoniae* [38]. In the case of bile solubility, the use of standardized bacterial cultures and measurements of changes in absorbance determined spectrophotometrically rather than by a visual assessment was shown to improve the test performance [39].

Automated systems, such as API® rapid ID 32 Strep or the VITEK® 2, are of a limited utility for identification of Mitis streptococci [40, 41]. The matrix-assisted laser desorption ionization time of flight mass spectrometry (MALDI-TOF MS) is an increasingly important technique for rapid identification of bacterial and fungal species [42] but the performance of this technique turned out to be problematic for some isolates belonging to the Mitis group [43, 44], although an update of reference database has recently improved the results of identification based on MALDI-TOF spectra [45].

## Immunological methods

Polysaccharide capsule, specified by the *cps* locus, represents the major virulence factor of pneumococci [46]. Identification of capsule type, i.e. serotyping is an important step confirming the identification and characterization of a pneumococcal isolate [38]. Specific antisera, currently available for 92 pneumococcal serotypes from the Statens Serum Institute (SSI, Copenhagen, Denmark; https://www.ssidiagnostica.com/neufeld-antisera/; 15th April 2020 date last accessed), are used in the Quellung reaction (named also Neufeld test or capsular swelling), which remains the “gold standard” for serotyping of pnemococci [38]. This test requires experienced personnel and a comprehensive panel of antisera, which in general limits its use to reference and research laboratories with a sufficiently large throughput of samples. Moreover, it was demonstrated that many representatives of *S. mitis*, *S. oralis* and *S. infantis* produce a capsular polysaccharide, which in some cases shows cross-reactivity in double immunodiffusion assays with antibodies recognizing several pneumococcal serotypes [47–49]. Importantly, however, tested Mitis group isolates producing capsular polysaccharide specific for serotypes 1 and 5 of *S. pneumoniae* reacted weakly or not at all in the classical Quellung test [48, 49], instead of demonstrating a distinct halo, typical for *S. pneumoniae* [14]. This difference is probably likely due to a much lower amount of polysaccharide produced by Mitis group streptococci other than *S. pneumoniae* [47]. Pneumotest-Latex (SSI) is a fast and easy to perform a test, however, also in this case, cross-reactions with *S. mitis* and *S. oralis* were occasionally observed for some reagent pools [50]. This test can also be applied directly to clinical samples [51]. Negative results of the Quellung test do not exclude that an isolate represents *S. pneumoniae* since non-serotypable (rough) pneumococci lacking capsule are observed and in fact, can be very common in some settings [52]. Such isolates may arise sporadically due to mutations in the *cps* locus or belong to certain lineages of *S. pneumoniae* devoid of the whole locus [53]. Moreover, new serotypes are constantly reported for *S. pneumoniae*, reaching at present 98 different ones [46]. Thus, currently available antisera may in some, presumably rare instances, yield ambiguous results in the case of pneumococci of yet uncharacterized serotypes. For more detailed information on immunological methods, the Reader may refer to the recent review on the identification of *S. pneumoniae* [54] that provides more detailed information on these issues.

## PCR-based methods of identification

The development of molecular methods and increasing knowledge of *S. pneumoniae* virulence determinants together with a better understanding of genetic diversity of pneumococci and other Mitis streptococci led to proposing several targets for PCR and real-time PCR in order to improve species identification among Mitis streptococci, especially concerning their questionable representatives. As PCR-based approaches do not require cultured microorganisms, they find also an application in the direct detection of pneumococci in clinical materials [55, 56], including polymicrobial samples [57–60]. Thus, the right selection of appropriate target gene(s) is of great importance. Ideally, such a target should be exclusively and ubiquitously present among *S. pneumoniae* and absent or sufficiently different in other Mitis streptococci to allow the clear discrimination of these groups.

A number of proposed genetic targets included *S. pneumoniae* virulence-associated genes. The *ply* gene, specifying the extracellular toxin pneumolysin, was one of the first DNA targets proposed to be used for the detection of pneumococci in clinical samples [61, 62]. However, its utility for identification of *S. pneumoniae* turned out to be limited [63] due to the presence of the *ply* gene also in other Mitis streptococci [26, 64–66]. The *lytA* gene, which encodes LytA, the major pneumococcal autolysin responsible for the solubilization of pneumococci in bile [67] was another gene proposed for detection purposes [68]. However, counterparts of *lytA* were found in other Mitis streptococci [64, 65]. Observed sequence differences between *lytA* alleles specific for pneumococci and other Mitis streptococci, the presence of a characteristic 6-bp deletion close to the *lytA* 3′ end in Mitis-specific variants of the gene in particular were the basis of a PCR-restriction fragment length polymorphism (PCR-RFLP) test [69] and a real-time PCR targeting this region [55]. While these assays are considered to have a very good species specificity for *S. pneumoniae* [38], variants typical for *S. pneumoniae* in isolates from the Mitis group, as well as a Mitis-specific allele in pneumococcus, were occasionally observed [24, 70]. The *psaA* gene, encoding pneumococcal surface antigen A (PsaA), which constitutes a manganese ABC-type transporter is commonly found among *S. pneumoniae* and was proposed as a target for PCR-based detection [71], showing good performance on collections of *S. pneumoniae* and other Mitis streptococci [55, 72, 73]. However, also this gene turned out to be present among some isolates of Mitis streptococci [26, 66, 74, 75]. Yet another proposed virulence-associated target, the *cpsA* (*wzg*) gene, which is typically the first gene in the *cps* operon, responsible for the biosynthesis of capsular polysaccharide [76], is absent among some non-serotypable pneumococci, as discussed above (see Immunological methods). In addition, the *cpsA* alleles of pneumococcal serotypes 25A, 25F and 38 are too divergent from typical pneumococcal *cpsA* genes [77] to yield positive results in this PCR, and such problems might also occur in the case of some novel serotypes. To additionally complicate identification issues, functional *cps* loci, including the conserved genes *cpsABCD*, were observed in a number of species of Mitis streptococci [47–49], suggesting a low utility of the *cpsA* target for discrimination between *S. pneumoniae* and other Mitis group representatives. Moreover, the similarity of some *cps* loci from the Mitis group streptococci to their counterparts in *S. pneumoniae* challenges the use of multiplex PCR for direct detection of pneumococcal serotypes, e.g. in carriage specimens [78]. Two genes, *piaA* and *piaB* encoding components of an ABC transport system essential for iron uptake, were described as specific for *S. pneumoniae* but these genes are absent in some pneumococcal isolates, which limits the sensitivity of the test [79–81]. Moreover, *piaA* was detected in some *S. mitis* and *S. pseudopneumoniae* [66] and recently *S. pseudopneumoniae* isolates positive for *piaB* have been reported [70].

Certain other genes, such as housekeeping genes and genes of unknown function were also proposed as targets for *S. pneumoniae* detection and identification. Sequence differences between variants of the *recA* gene, occurring in *S. pneumoniae* and other Mitis streptococci allowed designing a species-specific PCR [82], which could discriminate also *S. pseudopneumoniae* [83]. Although sequencing of the 16S rRNA gene, considered as the “gold standard” for identification of many bacterial species, has a limited utility for Mitis streptococci due to high similarity of 16S rRNA genes in this group [32], a cytosine at nucleotide position 203 in the 16S rRNA genes is considered specific for pneumococci, and sequence variants in this region of the gene can be distinguished using PCR-RFLP of the 16S rRNA genes [84], although the presence of mixed bases at this position was found for some *S. pseudopneumoniae* [26]. On the basis of genomic subtractive hybridization two loci of unknown function, Spn9802 and Spn9828 were proposed to be specific for *S. pneumoniae* [85, 86]. Later studies demonstrated that a PCR targeting spn9802 apart from *S. pneumoniae* also detects *S. pseudopneumoniae* but not *S. mitis* and *S. oralis* and thus can be used to distinguish these two groups [87]. Comparative genomic analyses of *S. pneumoniae* and *S. pseudopneumoniae* led to the identification of markers specific for these two species, respectively SPN0001/SP2020, encoding a GtnR-family transcriptional regulator, and SPS0002, specifying an osmosensitive potassium channel histidine kinase/response regulator KdpDE [88]. The specificity of both markers was close to 100%, with SPN0001/SP2020 found in a few non-pneumococcal strains and SPS0002 occasionally present in *S. pneumoniae* [70, 88].

Currently, it appears that a combination of a few assays, such as PCR-RFLP or real-time PCR specific for the 6-bp deletion in the 3′ end of *lytA*, together with detection of SPN0001/SP2020 [70], possibly supplemented with analysis of the presence of *piaA/piaB* could give more reliable results than a single-gene detection. Defining only one, “perfect” target, present among all *S. pneumoniae* strains, sufficiently conserved to design universal PCR primers and at the same time not found (or at least in a very divergent form) among any other Mitis group streptococci may be very difficult to achieve, if possible at all. This situation is associated with the common genetic ancestry of the Mitis group, relatively frequent HGT among streptococcal species, facilitated by their natural competence, together with a gene loss, resulting in populations with highly diverse gene content [89, 90]. In consequence, the core genome (genes common for each genome of a species) of *S. pneumoniae* is much smaller than the pangenome (a complete set of genes of a species) and many of these genes are shared with other Mitis streptococci, including determinants of factors playing the role in interactions with the host allowing a successful survival [91–94]. The recent genomic analyses of the Mitis group streptococci are discussed in more detail below (see Genomic studies in the identification of Mitis streptococci).

## Identification based on Sanger sequencing of single and multiple genes

The approach is based on analysis of sequence differences specific for various species, usually occurring in housekeeping genes, i.e. genes ubiquitously present among all Mitis streptococci, including *S. pneumoniae*. In comparison to PCR-based detection, Sanger sequencing is more time-consuming and represents a more expensive technology in terms of reagents and equipment. In addition, this approach requires highly qualified personnel and specialized software, although an increasing availability of commercial sequencing services makes it accessible for a growing number of laboratories. Both single-gene sequencing targets and multilocus schemes were proposed for the purposes of identification of *S. pneumoniae* and other Mitis streptococci.

The *sodA* gene encoding manganese-dependent superoxide dismutase, the *rpoB* gene encoding RNA polymerase beta-subunit and the *tuf* gene encoding elongation factor Tu were among the earliest proposed sequencing targets [95–98]. However, some later studies questioned their applicability for unambiguous identification, taking as reference results obtained with multiplex PCR, multilocus sequence analysis (MLSA, see below) or genomic analyses [16, 22, 87, 99, 100]. Other sequencing targets for identification purposes include *rnpB* (the RNase P RNA gene), *rpoA* (RNA polymerase alpha subunit gene), *recN* (recombination/repair protein gene), *gyrB* (DNA topoisomerase subunit B gene) and *rpsB* (the 30S ribosomal protein S2 gene) [80, 99, 101–103]; of these, *rnpB* and *gyrB* showed a good performance also in other studies [104].

Multilocus sequencing approaches, which target a few (usually seven) loci, occupying separate localizations on the bacterial chromosome, overcome to a significant extent the bias introduced by the gene exchange between *S. pneumoniae* and other Mitis streptococci. Two such schemes, multilocus sequence typing (MLST) [105] and multilocus sequence analysis (MLSA) [106], have been used in numerous studies. MLST, based on sequencing of seven housekeeping loci (*aroE*, *gdh*, *gki*, *recP*, *spi*, *xpt* and *ddl*) of *S. pneumoniae* is considered to unambiguously confirm the identification of an isolate as pneumococcus [107]. In MLST, after PCR amplification, products are sequenced and the identification of alleles and sequence types (STs) from allelic profiles is done using a large, web-accessible database (https://pubmlst.org/spneumoniae/; 15th April 2020 date last accessed), that currently contains over 15 thousand different STs and data for almost 50 thousand isolates of *S. pneumoniae* from all over the world. As a PCR-based technique, MLST can also be applied directly to clinical samples, such as cerebrospinal fluid [108]. While MLST operates on allelic profiles, MLSA is based on concatenated sequences of housekeeping loci that are used for the construction of dendrograms. The MLSA approach employing genes from the MLST schemes was used for species resolution of *Burkholderia* and *Neisseria* [109], while the identification of species within the Mitis group streptococci and other VGS seven different target loci (*map*, *pfl*, *ppaC*, *pyk*, *rpoB*, *sodA* and *tuf*) was selected [106]. More in the detail, sequences of loci belonging to an MLSA scheme, obtained for an analysed isolate are concatenated in a defined order, aligned with corresponding reference sequences and the alignment is used in phylogenetic clustering. The position of an isolate in the resulting tree is then examined for assigning a particular species. Unfortunately, the website described in the original paper on MLSA of VGS and dedicated for this purpose (http://www.eMLSA.net) appears to be no longer active (as of 15 April 2020). The MLSA scheme for VGS has proven its utility in a number of studies [19, 21, 22, 41, 99, 110]. This method showed a very good concordance with the results of core genome clustering [2, 26, 100] and species identification based on sequencing with the minION napore [41].

## Genomic studies in the identification of Mitis streptococci

The recent advances in whole-genome sequencing (WGS), including the availability of software for analysis of genomic data, have opened new possibilities for taxonomy and identification of Mitis streptococci. Bioinformatics tools have been developed for purposes of species identification, such as Kraken, which allows fast assigning taxonomic labels from short-read sequencing data, including metagenomic samples [111]. Such a metagenomic approach was recently applied for the detection of pathogen DNA in cerebrospinal fluid samples from patients with pneumococcal meningitis [112]. Ribosomal MLST (rMLST) was designed as a universal identification tool for bacteria, which indexes variation of the 53 genes of bacterial ribosomal proteins [113]. For identification of species and a ribosomal sequence type (rST), assembled genomic sequences are submitted to a specialized database (https://pubmlst.org/rmlst/; 20th April 2020 date last accessed) for a query. The results of rMLST turned out to be in very good agreement with the core genome-based clustering of *S. pneumoniae*, *S. mitis* and *S. pseudopneumoniae* [26].

Clustering based on the variability of genes belonging to the core genome of Mitis streptococci, i.e. genes present among all studied strains, has proven to be the most informative and reliable approach for analyses of phylogenetic and taxonomic relationships among Mitis streptococci [2, 91, 100]. These investigations also allowed re-assigning species of several isolates of Mitis streptococci, in which genomic data were deposited in GenBank [2], demonstrating the utility of core genome-based studies for identification. The MinION nanopore sequencer developed by Oxford Nanopore Technologies (ONT) provides long sequencing reads in real time and its small size allows performing analyses also in the field conditions. Sequencing results can be analysed using either Kraken or the “What’s in my Pot?” (WIMP) analysis pipeline [114]. The analysis of data from the MinION nanopore sequencer with WIMP and Kraken2 provided reliable identification of clinical isolates of Mitis streptococci [41], opening new possibilities in the detection and identification of these microorganisms.

Although genomic analyses are currently limited to highest-level reference centres and research laboratories, the importance and the popularity of this approach will certainly grow in the near future, with the presumable continuation of diminishing costs of sequencing and availability of more user-friendly software. Increasing amounts of genomic data for streptococci will aid the re-evaluation of known PCR and sequencing targets as well as identification of new ones for quick and cost-effective diagnostics, such as already demonstrated for SPN0001 and SPS0002 [88], discussed above. A recent comparative analysis [92] performed on 60 genomes of *S. pneumoniae*, *S. mitis*, *S. pseudopneumoniae*, *S. oralis* and *S. infantis* followed by a screening of over 7500 available streptococcal genomes allowed the identification of 224 genes present in at least 85% of *S. pneumoniae* genomes but absent from 80% or more genomes of *S. mitis*, and thus postulated to be associated with virulence of pneumococcus, as opposed to the commensal lifestyle of *S. mitis*. Of these 224 genes, 49 ones, which were totally absent from *S. mitis*, *S. pseudopneumoniae* and *S. oralis*, included genes encoding surface-located proteins PspA, CbpA and “Xisco”, the iron uptake operon *piaABCD*, genes involved in carbohydrate metabolism and others. Pangenomic analysis of 32 genomes of *S. pneumoniae*, 36 genomes of *S. mitis* and 13 genomes of *S. pseudopneumoniae* followed by a validation of results on larger panels of streptococcal genomes revealed ten genes, residing in five loci, specific solely for *S. pneumoniae* and nine genes unique for *S. pseudopneumoniae* [94]. Four of loci characteristics for *S. pneumoniae*, including the *piaABCD* operon, the locus containing SP2020 and two single genes, encoding a putative ABC transporter and a hypothetical protein, were common with the set of 49 genes from the previously mentioned study [92]. Additional analyses of six selected genes, longer than 500 bp, considered specific for *S. pneumoniae* and proposed as PCR targets revealed the presence of four of them in a few genomes of *S. pseudopneumoniae* consistent with the need to use more than a single marker for identification purposes [94].

## Conclusions

The correct identification of species belonging to the Mitis group of streptococci is important for clinical practice, epidemiology and studies on the biology of these organisms. Several molecular approaches have been proposed over time to supplement traditional microbiological methods. The performance of PCR-based methods, targeting a single gene varied considerably, mostly due to the presence of counterparts of some of these “pneumococcal” genes in other Mitis streptococci, in many cases presumably associated with the high frequency of HGT. The circulation of genuine pneumococci lacking certain target genes represents another issue that complicates PCR-based detection and identification. Using the combination of a few selected targets significantly increases the probability of correct identification by PCR, which represents a fast and cost-effective method, available for many laboratories. Multilocus sequencing (such as the MLST and MLSA approaches) and genomic sequencing provide a clear distinction among species of the Mitis group and it can be expected that in the near future the ongoing advances in WGS and downstream data analysis will make such approaches accessible for an increasing number of laboratories. Genomic studies have already greatly improved our understanding of relationships within the group of Mitis streptococci and they will also aid to better define consensus targets for fast, cost-effective and specific detection of *S. pneumoniae* and other Mitis streptococci.
